# Antimicrobial resistance in pathogenic aerobic bacteria causing surgical site infections in Mbarara regional referral hospital, Southwestern Uganda

**DOI:** 10.1038/s41598-019-53712-2

**Published:** 2019-11-21

**Authors:** Derick Hope, Lucas Ampaire, Caesar Oyet, Enoch Muwanguzi, Hillary Twizerimana, Richard Onyuthi Apecu

**Affiliations:** 1 Department of Medical Microbiology, Mbarara University of Science and Technology, P.O. BOX 1410, Mbarara, Uganda; 2 Department of Medical Laboratory Sciences, Mbarara University of Science and Technology, P.O. BOX 1410, Mbarara, Uganda; 3 Obstetrics and Gynecology Unit, Mbarara Regional Referral Hospital, P.O. BOX 40, Mbarara, Uganda; 4 School of Allied Health, Clarke International University, P.O. BOX 7782, Kampala, Uganda

**Keywords:** Antimicrobial resistance, Bacterial infection

## Abstract

Surgical site infections (SSI) remain a common postoperative complication despite use of prophylactic antibiotics and other preventive measures, mainly due to increasing antimicrobial resistance. Here, we present antimicrobial resistance rate of bacteria isolated in clinical cases of SSI. A hospital based descriptive cross sectional study was conducted on 83 consented postoperative patients with clinical SSI. Data on patients was obtained using structured data collection form. Two swabs were collected aseptically from each patient. Bacteriological culture examination and identification was done following standard microbiological techniques. Antibiotic susceptibility test was done by Kirby-Bauer disc diffusion method. Gram negative bacteria (GNB) were predominant (65.59%) with the dominant being *Klebsiella* species (29.03%). Overall 86% of aerobic bacteria isolated were multidrug resistant (MDR) where 65.63% and 96.72% of Gram positive and Gram negative isolates were MDR respectively. All the isolates with exception of *Enterococci* species were resistant to ampicillin. GNB showed high resistance to ceftriaxone, sulfamethoxazole/trimethoprim and gentamicin. All the isolated *Klebsiella* spp were MDR. *S. aureus* were all resistant to oxacillin. The isolation rate was higher in emergency, males and dirty wounds in relation to nature of surgery, gender and class of surgical wound respectively. These findings necessitate judicious antibiotic use and calls for surveillance of SSIs periodically as well as strict adherence to good sanitation practice to reduce spread of drug-resistant pathogens.

## Introduction

Surgical site infection (SSI) is an infection occurring within thirty (30) days after surgery or after a year in case of an implant^1^, due to contamination of the surgical site (incision) by microorganisms. Contamination of the surgical site by microorganisms occurs during surgical procedure or postoperative wound care settings. The surgical site can be contaminated from sources within the patient such as patient flora, remote infection; or external sources such as surgical personnel, physical environment and ventilation, and tools/equipment/materials in the operation theatre^2,3^.

Despite use of prophylactic antibiotics pre- and postoperatively and other preventive measures such as improved operating room ventilation, sterilization methods, use of barriers, surgical technique, SSIs still remain a burden to postoperative patients^4^. This has majorly been attributed to increasing emergence of antimicrobial resistance^5,6^ due to irrational use of antibiotics. This inappropriate use of antimicrobials increases selection pressure favoring emergence of pathogenic drug resistant bacteria.

There is no data on the global epidemiology of SSI due to lack of standardized diagnosis, absence of surveillance and notification system in many developing countries^4,7^. SSI is the leading cause of all health-care associated infections (HAI) in developing countries^7,8^. The cumulative incidence of SSI in Africa varies from 2.5–30.9% as reported in a systematic review^9^. SSI causes a marked health burden in terms of patient morbidity and mortality, prolonged hospitalization, increased cost of treatment to patients, increased resistance of microorganisms to antimicrobials, and a massive additional financial burden for health systems^7,10^. Information on burden of HAI is scanty in developing countries. In Uganda, it is reported that about 10% of the surgical procedures become septic^11^. The incidence of SSI at Mbarara regional referral hospital (MRRH) in 2015 was revealed to be 16.4%^12^. Many studies in Uganda report the most common bacterial pathogens as *E. coli, Klebsiella species, Acinetobacter species, P. aeruginosa, S. aureus, Enterococcus species, Proteus mirabilis* and *Enterobacter species*^6,12,13^. The emergence of antimicrobial resistant strains of hospital pathogens has also presented a challenge in the provision of good quality in-patient care. A study about antimicrobial resistance in bacterial pathogens causing SSI conducted in a national hospital, Tanzania revealed that 63% of the isolates were multidrug resistance (MDR)^14^. While in Uganda, a similar study conducted in the national hospital showed that MDR was reported to be 78% among the bacterial isolates of SSI^6^. At MRRH, number of patients with clinical SSI observed in surgical and gynecology wards is increasing yet data on the bacterial isolates causing SSI and their antimicrobial susceptibility pattern is limited. Therefore, the aim of this study was to determine the bacterial pathogens from hospital acquired surgical site infection and determine their antimicrobial resistant patterns among postoperative patients at MRRH.

## Results

### Demographic and clinical characteristics

A total of 83 wound swabs were collected from patients with clinical SSIs. Tables 1 and 2 present the demographic and clinical information. Obstetrics/post-natal ward represented 32.53% of the patients. The age ranged from 6–75 years with mean of 26.51 ± 13.56. Majority of the patients were females (65.06%). The most common surgical procedure was caesarian section (45.78%) while emergency surgery was the most common type of surgery (59.04%). Less than 50% (45.78%) of the wounds were classified as clean type whilst majority of the clinical SSIs noted were deep incisional (50.6%). Majority of the patients (54.22%) had operations done on the same day of admission while over 50% developed infection within the first week of operation. All the study participants were subjected to antibiotic prophylaxis and the mostly used antibiotics included ampicillin-cloxacillin, metronidazole, ceftriaxone, ciprofloxacin and gentamycin.

**Table 1:** Socio-demographic and Clinical characteristics.

Variable		Ward/Department				Total	
		Gynecology	Obstetrics	Surgical	Orthopedics	No.	%
1. Age (years)	1–10	—	—	5	—	5	6.02
	11–20	4	9	9	7	29	34.94
	21–30	9	16	2	5	32	38.55
	31–40	—	2	4	3	9	10.84
	41–50	—	—	—	2	2	2.41
	51–60	—	—	—	2	2	2.41
	61–70	—	—	—	2	2	2.41
	71–80	—	—	—	2	2	2.41
	**Total**					**83**	**100**
2. Sex	Female	13	27	11	3	54	65.06
	Male	—	—	9	20	29	34.94
	**Total**					**83**	**100**
3. Nature of surgery	Elective	2	—	18	14	34	40.96
	Emergency	11	27	2	9	49	59.04
	**Total**					**83**	**100**
4. Class of surgical wound	Clean	7	11	7	13	38	45.78
	Clean-cont.^1^	4	10	8	5	27	32.53
	Contaminated	2	4	—	5	11	13.25
	Dirty/Infected	—	2	5	—	7	8.43
	**Total**					**83**	**100**
5. Type of SSIs	Superficial	4	16	5	9	34	40.96
	Deep	9	11	8	14	42	50.60
	Organ/Space	—	—	7	—	7	8.43
	**Total**					**83**	**100**
6. Surgical Procedure	C-section^2^	13	25	—	—	38	45.78
	SD^3^	—	—	11	14	25	30.12
	Laparatomy	—	2	9	—	11	13.25
	ORIF^4^	—	—	—	4	4	4.82
	Others	—	—	—	5	5	6.02
	**Total**					**83**	**100**

**Key: 1**-Clean-contaminated, **2**- Caesarian section, **3**-Surgical debridement, **4**-Open reduction and internal fixation.

**Table 2:** Duration of pre-operative stay and post-operative presentation of SSIs.

Days		Wards/Department				Total	
		Gynecology	Obstetrics	Surgical	Orthopedics	No.	%
1. Pre-operative^1^	0	9	18	4	14	45	54.22
	1	4	8	11	5	28	33.73
	2	—	1	—	2	3	3.61
	3	—	—	—	—	—	—
	4	—	—	5	2	7	8.43
	**Total**					**83**	**100**
2. Post-operative presentation of clinical SSI^2^	1	—	—	—	—	—	—
	2	—	5	2	5	12	14.46
	3	1	9	2	—	12	14.46
	4	2	2	5	—	9	10.84
	5	—	2	2	2	6	7.23
	6	—	2	5	2	9	10.84
	7	4	—	—	—	4	4.82
	8	—	—	—	—	—	—
	9	2	—	—	—	2	2.41
	≥10^3^	4	7	4	14	29	34.94
	**Total**					**83**	**100**

**Notes: 1-**From admission to operation, **2**-From operation to the first day of presentation of SSIs, **3**-≥10 but within a month.

### Laboratory results

#### Culture results

**Figure 1:**
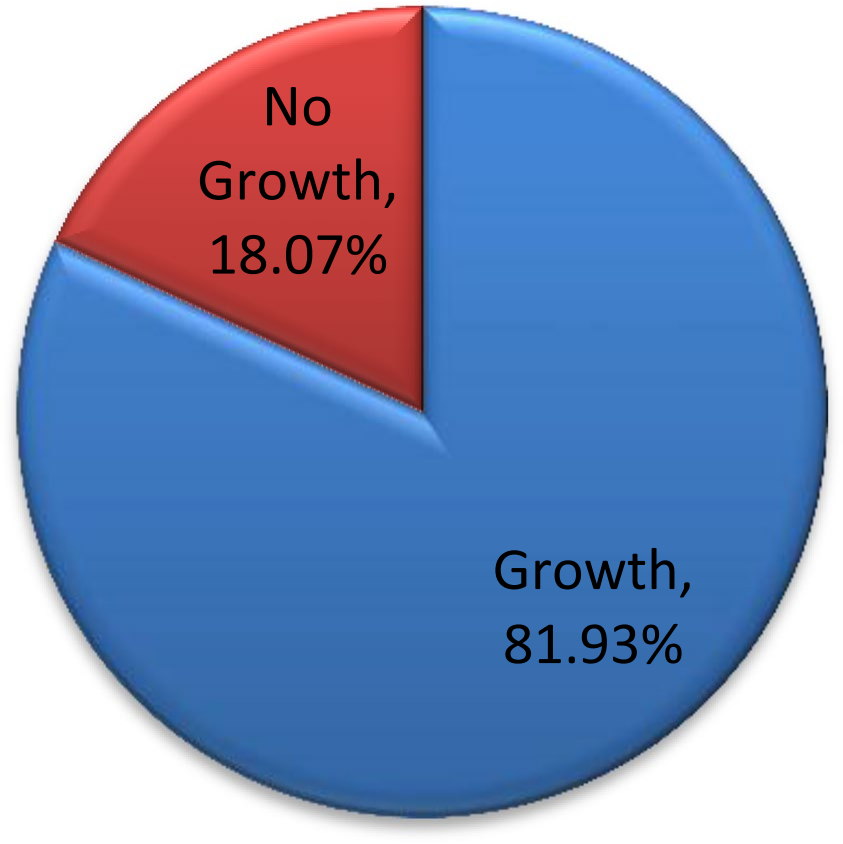
Proportion of culture positivity. Proportion of patient samples that have grown/not grown on culture expressed as a percentage of total number of samples.

Out of 83 samples, 81.93% were culture positive aerobically for bacteria (Fig. 1). Among the positive cultures, bacteria and pus cells were seen in 66.18% and 58.82% of the samples and not seen in 33.82% and 41.18% of the samples respectively (Table 3). While among the negative cultures, bacteria and pus cells were seen in 13.33% and 20% of the samples and not seen in 86.67% and 80% of the samples respectively (Table 3). Culture positivity/Isolation rate was higher for males (86.21%) compared to females (79.63%), (p-value = 0.354) and higher for emergency (89.8%) compared to elective (70.59%), (p-value = 0.0001) whereas 95.24% of deep incisional SSIs (p-value = 0.0026) and 100% of dirty surgical wounds (p-value = 0.0002) were culture positive (Table 4).

**Table 3:** Direct microscopy in relation to culture results.

Recovery status	Initial Gram stain microscopy			
	Bacteria		Pus cells	
	Seen	Not seen	Seen	Not seen
Growth, n = 68	45 (66.18%)	23 (33.82%)	40 (58.82%)	28 (41.18%)
No growth, n = 15	2 (13.33%)	13 (86.67%)	3 (20%)	12 (80%)

**Table 4:** Isolation rate by demographic and clinical characteristics of patients.

Characteristic		Number tested	Growth	P-value
1. Sex	Female (%*)	54 (65.06)	43(79.63)	0.354
	Male (%*)	29 (34.94)	25(86.21)	
	**Total**	**83**	**68**	
2. Type of SSIs	Superficial (%*)	34 (40.96)	23(67.65)	0.0026
	Deep (%*)	42 (50.60)	40(95.24)	
	Organ (%*)	7 (8.43)	5(71.43)	
	**Total**	**83**	**68**	
3. Nature of surgery	Elective (%*)	34 (40.96)	24(70.59)	0.0001
	Emergency (%*)	49 (59.04)	44(89.80)	
	**Total**	**83**	**68**	
4. Class of surgical wound	Clean (%*)	38 (45.78)	29(76.32)	0.0002
	Clean-cont^#^ (%*)	27 (32.53)	22(81.38)	
	Contaminated(%*)	11 (13.25)	10(90.91)	
	Dirty (%*)	7 (8.43)	7(100)	
	**Total**	**83**	**68**	
5. Age (%*)	1–10	5 (6.02)	0 (0)	0.0597
	11–20	29 (34.94)	25(86.21)	
	21–30	32 (38.55)	28(87.5)	
	31–40	9 (10.84)	7 (77.78)	
	41–50	2 (2.41)	2 (100)	
	51–60	2 (2.41)	2 (100)	
	61–70	2 (2.41)	2 (100)	
	71–80	2 (2.41)	2 (100)	
	**Total**	**83**	**68**	

*%- Percentage of growth/no growth independently for each characteristic. ^#^Clean-contaminated.

**Figure 2:**
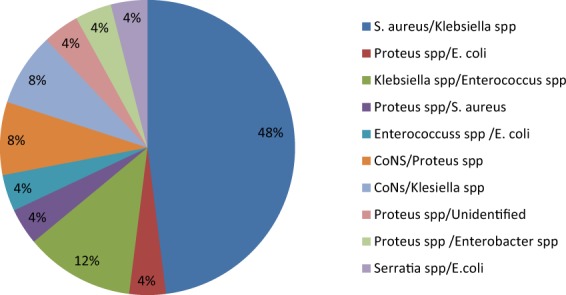
Patterns of isolates in polymicrobial infections. Combination of bacterial pathogens isolated on culture from wound swab collected at one surgical site from a participant.

**Figure 3:**
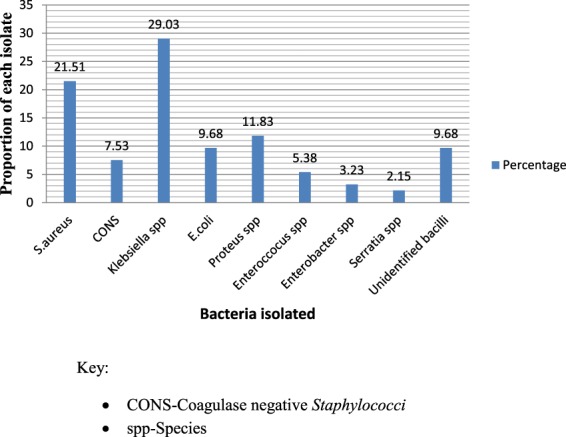
The proportion of bacterial isolates. The proportion of bacterial species isolated expressed as a percentage of the total number of isolates.

**Figure 4:**
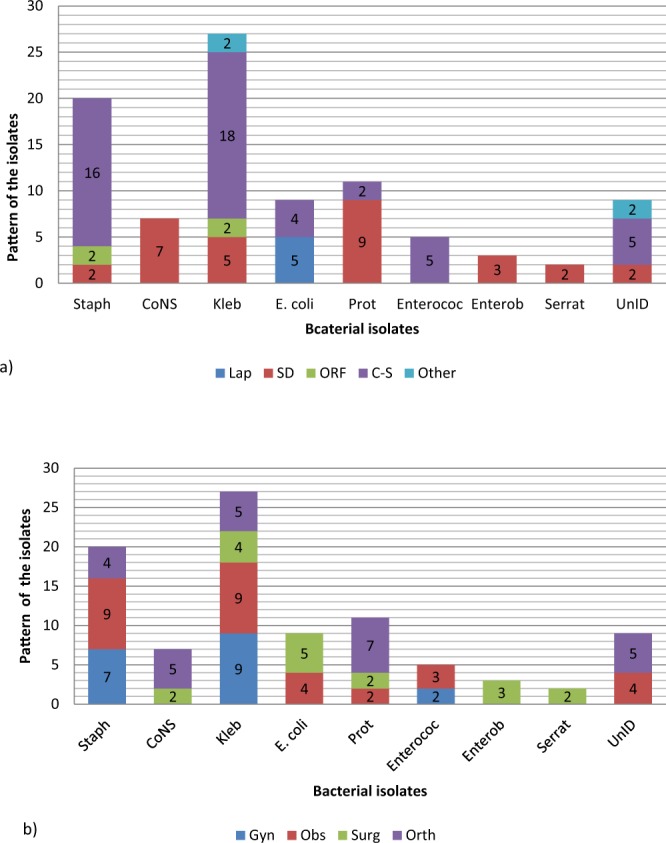
Pattern of bacterial isolates in relation to surgical procedure (**a**) and ward (**b**). The distribution of the different species of bacteria according to surgical procedure and ward. **Key;** Staph-*Staphylococcus aureus*, CONS-Coagulase negative *Staphylococci*, Kleb-*Klebsiella* species, E. coli-*Escherichia coli*, Prot-*Proteus* species, Enteroc-*Enterococcus* species, Enterob-*Enterobacter* species, Serrat-*Serratia* species, UnID-unidentified Gram negative bacilli. Gyn-Gynecology, Obs-Obstetrics, Surg-Surgical, Orth-Orthopedics; Lap-Laparatomy, SD-Surgical debridement, ORF-Open reduction and internal fixation, C-S – Caesarian section, other-Other procedures.

Out of the culture positive samples, a total of 93 bacterial isolates were recovered aerobically where 43 (63.24%) were single/pure isolates and 25 (36.76%) being dual (mixed) isolates, of which *S. aureus* and *Klebsiella spp* was the commonest (48%) combination (Fig. 2). The Gram negative bacteria were predominant, 61 (65.59%) compared to Gram positive bacteria, 32 (34.41%). The frequently isolated bacteria included *Klebsiella* species (29.03%), *Staphylococcus aureus* (21.51%), *Proteus* species (11.83%) and *Escherichia coli* (9.68%). Others included Coagulase negative *Staphylococci* species (CoNS) (7.53%), *Enterococci* species (5.38%), *Enterobacter* species (3.23%) and *Serratia* species (2.15%) (Fig. 3). Unidentified Gram negative bacilli represented 9.68% of the isolates. *E. coli* and *Klebsiella species* were the prevalent isolates in organ and deep incisional SSIs respectively while *S. aureus* and *Klebsiella species* were predominant in superficial incisional SSI (p-value < 0.000, χ^2^ = 58.543). In relationship to the surgical procedure, *Klebsiella* species and *Staphylococcus aureus* were the dominant isolates in C-section while *E. coli* and *Proteus species* were the commonest isolates in laparatomy and surgical debridement respectively (Fig. 4a). *Klebsiella* species and *Staphylococcus aureus* were the dominant isolate in obstetrics and gynecology wards whereas *Proteus* species and *E. coli* were the most common in orthopedics and surgical ward respectively (Fig. 4b).

#### Antimicrobial Resistance pattern among the isolates

Generally, 97% of the Gram positive bacteria were resistant to at least one class of the drugs used (only one isolate of *Enterococcus species* was sensitive to all antibiotics) with 65.63% showing MDR, defined as non-susceptibility to three or more of the antibiotics tested (Tables 5 and 6). Most resistance was expressed against ampicillin (84.38%), where with exception of *Enterococci* species all were resistant to ampicillin. The Gram positive bacteria responded differently to other antibiotics but in general showed high resistance to oxacillin (81.25%), ceftriaxone (78.13%) and moderate resistance to sulfamethoxazole/trimethoprim (56.25%), gentamycin (56.25%), erythromycin (65.63%) and low resistance to ciprofloxacin (37.5%). All *Staphylococcus aureus* isolated were resistant to oxacillin and ampicillin; showed moderate resistance to ciprofloxacin (50%) and high resistance to other antibiotics tested (Table 5). 45% of the *S. aureus* isolated were resistant to all the antibiotics tested. The CoNS were all resistant to ampicillin; sensitive to sulfamethoxazole/trimethoprim, ciprofloxacin and gentamicin and had low to moderate resistance to other antibiotics (Table 5). The *Enterococci* species showed low to moderate resistance to the antibiotics tested except for ampicillin where all were sensitive (Table 5).

**Table 5:** Resistant patterns of bacterial isolates.

Isolates	Antibiotics tested						
	CRO	SXT	E	AM	CIP	OX	GM
**Gram positive bacteria**							
*S. aureus* (%), n = 20	18 (90)	15 (75)	17 (85)	20 (100)	10 (50)	20 (100)	15 (75)
CoNS (%), n = 7	4 (57.14)	0	2 (28.57)	7 (100)	0	3 (42.86)	0
*Enterococci ssp* (%), n = 5	3 (60)	3 (60)	2 (40)	0	2 (40)	3 (60)	3 (60)
Total (%), n = 32	25 (78.13)	18 (56.25)	21(65.63)	27 (84.38)	12 (37.5)	26 (81.25)	18 (56.25)
**Gram negative bacteria**							
*Klebsiella spp* (%), n = 27	23 (85.19)	22 (81.48)	NT	27 (100)	15 (55.56)	NT	22 (81.48)
*Proteus spp*(%), n = 11	9 (81.82)	10 (90.91)	NT	11 (100)	7 (63.64)	NT	9 (81.82)
*E. coli* (%), n = 9	9 (100)	9 (100)	NT	9 (100)	7 (77.78)	NT	7 (77.78)
*Enterobacter* spp (%), n = 3	3 (100)	3 (100)	NT	3 (100)	2 (66.67)	NT	2 (66.67)
*Serratia* spp (%), n = 2	2 (100)	2 (100)	NT	2 (100)	2 (100)	NT	2 (100)
UGN (%), n = 9	8 (88.89)	8 (88.89)	NT	9 (100)	6 (66.67)	NT	7 (77.78)
Total (%), n = 61	54 (88.52)	54 (88.52)	NT	61 (100)	39 (63.93)	NT	49 (80.33)

**Key:** CRO-ceftriaxone, SXT-sulphamethoxazole/trimethoprim (cotrimoxazole), E-erythromycin, AM-ampicillin, CIP-ciprofloxacin, OX-oxacillin, GM-gentamicin, CoNS-coagulase negative *Staphylococci*, Spp-species, UGN-unidentified Gram negative bacilli, NT-not tested.

**Table 6:** Multiple drug resistant patterns of the isolated bacteria.

	R_0_	R_1_	R_2_	R_3_	R_4_	R_5_	R_6_	R_7_
*S. aureus* (n = 20)	—	—	3 (15%)	—	—	5 (25%)	3 (15%)	9 (45%)
*Klebsiella spp* (n = 27)	—	—	—	9 (33.33%)	11 (40.74%)	7 (25.93%)	—	—
*Proteus spp* (n = 11)	—	—	2 (18.18%)	3 (27.27%)	4 (36.36%)	2 (18.18%)	—	—
*E*. *coli* (n = 9)	—	—	—	—	4 (44.44%)	5 (55.56%)	—	—
CoNS (n = 7)	—	3 (42.86%)	4 (57.14%)	—	—	—	—	—
*Enterococci spp* (n = 5)	1 (20%)	—	—	2 (40%)	2 (40%)	—	—	—
*Enterobacterspp* (n = 3)	—	—	—	2 (66.67%)	1 (33.33%)	—	—	—
*Serratia spp* (n = 2)	—	—	—	—	—	2 (100%)	—	—
UGN (n = 9)	—	—	—	5 (55.56%)	4 (44.44%)	—	—	—
Total (n = 93)	1 (1.08%)	3 (3.23%)	9 (9.68%)	21 (22.58%)	26 (27.96%)	21 (22.58%)	3 (3.23%)	9 (9.68%)

Key: CoNS-coagulase negative staphylococci; spp-Species; R_0_-sensitive to all antibiotics tested; R_1_, R_2_, R_3_, R_4_, R_5_, R_6_, R_7_-Resistant to one, two, three, four, five, six, seven antibiotics respectively. Ssp-Species.

Among the Gram negative bacteria, 96.72% were multidrug resistant and 26% were resistant to all the antibiotics tested (Tables 5 and 6). All the Gram negative isolates (100%) were resistant to ampicillin. The isolates in general showed moderate resistance to ciprofloxacin (66.67%) and high resistance to ceftriaxone (88.89%), sulfamethoxazole/trimethoprim (88.89%) and gentamicin (77.78%). 25.93% of *Klebsiella* species were resistant to all antibiotics, and showed high resistance to ceftriaxone (85.19%), sulfamethoxazole/trimethoprim (81.48%), gentamicin (81.48%) and moderate resistance to ciprofloxacin (55.56%). *Proteus* species showed high resistance to ceftriaxone (81.82%), sulfamethoxazole/trimethoprim (90.91%), gentamicin (81.82%) and moderate resistance to ciprofloxacin (63.64%). All the isolates of *Escherichia coli* were resistant to at least 4 antibiotics, of which 55.56% were resistant to all antibiotics and showed high resistance to all the antibiotics tested (Tables 5 and 6). The species of *Enterobacter* isolated were all resistant to ceftriaxone, sulfamethoxazole/trimethoprim and showed moderate resistance to ciprofloxacin (66.67%) and gentamicin (66.67%). The isolated *Serratia* species were resistant to all the tested antibiotics. The unidentified Gram negative bacilli showed moderate resistance to ciprofloxacin (66.68%) and high resistance to the other antibiotics tested (77.78–100%) (Table 5).

Table 6 summarizes multiple drug resistance shown by the isolates. 1.1%, 3.2% and 86.0% of the isolates showed total sensitivity, resistance to a single antibiotic agent and multidrug resistance respectively to the antibiotics tested. 45% of the *Staphylococcus aureus* and 25.93% of the *Klebsiella species* were resistant to all the antibiotics tested (Table 6).

## Discussion

The culture positivity of 81.93% in our study was higher compared to the isolation rate of 68.8% in a referral hospital in Uganda^6^, 71% in Ethiopia^15^ and 60.6% in Nepal^16^. The high proportion in the present study was found to be the result of persistent antimicrobial resistant pathogenic bacteria as discussed later. The proportion of culture positivity was comparatively high at 90% in Tanzania^14^ and 96% in India^17^. Generally, the possible variation in culture positivity could be attributed to differences in the infection control/prevention practices and differences in the population studied (comorbid illnesses, sex, age).

In this study there was 18.07% culture negativity, suggesting possibility of susceptible aerobes or anaerobe as shown by 13.33% bacteria seen on direct smear among the culture negatives. Anaerobic culture was not done in this study. There is also possibility infection by other microbes other than bacteria.

The isolation rate was not significantly affected by gender (p-value = 0.354), being higher for males (86.21%) compared to females (79.63%) which agrees with findings from other studies with males having more rates; 80.2% males and 57.7% females, p < 0.001^15^, and 81.3% males and 64.9% females^6^. This could be due to a number of reasons including males being more exposed to risk factors like cigarette smoking which has been found to be associated with increased SSIs rate^1,18^, males being mostly accident cases with increased colonisation of exposed wound and differences in adherence to treatment. Isolation in the present study was found to be higher in emergency procedures compared to elective (p < 0.0001) which complies with related studies^17,19^. The possible reason behind this could be due to the fact that emergency surgeries being life saving procedures might compromise on the level of aseptic techniques employed and possibility of prolonged complicated emergency cases which predispose to inoculation of pathogenic microorganisms in surgical site. Isolation rate in the current study was found to be more in dirty wounds, followed by contaminated, clean-contaminated and clean (p < 0.0002). This concurs with other studies^17,19,20^. This could be explained by the level of microbial load which is higher in contaminated wounds increasing chances of isolation.

A total of 93 aerobic bacteria were isolated where more pure isolates (63.24%) were recovered than mixed (36.76%) which was in consistence with similar studies^6,14–16^, however, it was in contrast with a study in Italy where more mixed isolates were recovered compared to pure ones^21^. Class of surgical wound plays a role in the purity of the isolates where clean procedures are associated with monomicrobial isolates while contaminated and dirty wounds are associated with polymicrobial isolates^17^. Majority of the surgeries in this study were clean.

The preponderance of Gram negative bacteria in the current study was in agreement with findings from neighboring Tanzania and Ethiopia^14,22^. This could be attributed to diverse habitat of Gram negative bacteria including inanimate surfaces in hospitals, multidrug resistant patterns portrayed and possible contamination from intestinal tract during surgery. *Klebsiella* species was the predominant isolate which contrasts with similar studies that reported *S. aureus* as the predominant isolate^13,15,19,23^. *Klebsiella* species have been reported common contaminants in operating room air and fomites including medical equipments in hospitals^24^. Others studies reported *P*. *aeruginosa* (not isolated in this study) as the dominant isolate^14,16^. Species and proportion of isolated bacteria vary according to the place and year. The posible reason for variation in the species isolated could be attributed to differences in aseptic techniques followed, diverse geographical distribution of causative agents, varied resistant patterns of the bacterial isolates in question, and difference in the surgical procedures performed among other reasons. When internal organs are resected through the abdomen, the causative agents included the normal Gram negative flora of the gut and in clean procedures, exogenous bacteria or skin colonizers are recovered^17^.

In this current study, *in vitro* antibiotic susceptibility to the commonly used drugs showed that the bacterial isolates responded differently to the tested antibiotics. With exception of *Enterococci* species all Gram positive bacteria were resistant to ampicillin. The reason behind this could be the irrational use of ampicillin, which was one of the most used antibiotics for empiric prophylaxis. Similar results of complete resistance (100%) by Gram positives to ampicillin were reported in India^17^ and North West Ethiopia^25^. Ciprofloxacin was seemingly the drug of choice for the Gram positives according to our study. All *S. aureus* showed 100% resistance to ampicillin and oxacillin and high resistance to other antibiotics tested except ciprofloxacin. Similar results of resistance to ampicillin were observed elsewhere^13,17,25^. However in contrast, sensitivity to oxacillin was observed in related studies, 96%^16^ and 33%^17^. *S. aureus* in this study showed high resistant to gentamicin (75%) which conquered with 70% resistance in a similar study^17^, however it contrasted with the 87.5% sensitivity as reported in a similar study in Uganda^13^. Variation in the susceptibility pattern could be attributed to difference in rational use of antibiotics. All the Gram negative bacteria isolated in this study were resistant to ampicillin, which was in concurrence with a study in India^17^ and showed high resistance to other antibiotics tested except ciprofloxacin. Other studies in Uganda and elsewhere reported a high resistance (90–97%) by the Gram negative bacterial isolates to ampicillin^13,15,19,25^. *Klebsiella species* showed high resistance to ceftriaxone, trimethoprim-sulphamethoxazole, ampicillin, gentamicin and moderate resistance to ciprofloxacin and it was in agreement with similar studies in Ethiopia and India^15,17^. But in contrast, a study in Uganda reported that *Klebsiella* species had 100% sensitivity to gentamicin^13^. *E. coli* showed high resistance (78–100%) to antibiotics tested, similar to findings from a study in India^17^ while other studies show moderate resistance (40–60%)^15,25^. Among the *Proteus species*, there was moderate resistance to ciprofloxacin but high resistance to other antibiotics tested, which was in contrast to study in Ethiopia^15^ where there was low resistance to trimethoprim-sulphamethoxazole, gentamicin, ceftriaxone and ciprofloxacin. In this study MDR was observed to be 86% which was comparable to the 78% reported in Uganda^6^ but much higher than the 63% described in Tanzania^14^. Microorganisms from the hospital environment are exposed to various antimicrobial agents and have been shown to express high antimicrobial resistance due to selection pressure^26,27^ hence posing difficulty in the therapeutic management of such hospital-acquired infections. Resistance to the commonly used antibiotics could also be attributed to the injudicious use of the antibiotics by clinicians without evidence of causative agent and antibiogram, and misuse resulting from self-treatment with the readily available and cheap over-the-counter antibiotics. The high antibiotic resistance in this study implies that the available antibiotics might be rendered useless if immediate action is not taken and calls for stringent measures on antimicrobial stewardship as well as search of new antibiotics.

We certainly had limitations in the study including inability to isolate causative agents of SSI other than aerobic bacteria such as strict anaerobes and fungi due to inadequate funds which could have increased the positivity rate. We only included in our susceptibility test the commonly used antibiotics in the study site.

In conclusion, SSI is still a major problem in postoperative patients in the study site. There was an alarming MDR rate of 86% among the bacterial isolates and high resistance to the commonly used antibiotics. We strongly recommend that antibiotic therapy should be guided by antimicrobial susceptibility patterns. We recommend surveillance of SSIs periodically including incidence, aetiology, antibiotic susceptibility profile and source of infection. We suggest a preoperative rectal swab to detect colonization with MDR bacteria in order to isolate affected patients and avoid wasteful usage of antibiotics. Finally, we recommend strict adherence to good sanitation practice including thorough hand washing, disinfection of inanimate objects and other infection control measures so as to minimize the spread of MDR strains of bacteria.

## Methods

### Study design and setting

This descriptive cross-sectional study was conducted in surgical, obstetrics/post-natal, gynecology, and orthopedics wards of MRRH. MRRH serves as a referral hospital for southwestern Uganda and it is located about 265 Km by road southwest of Kampala, the capital of Uganda. The hospital also receives patients from neighboring countries like Democratic republic of Congo, Rwanda, and Tanzania.

### Sampling and data collection

The study population consisted of postoperative patients in the study wards with clinical SSI within 30 days of operation. A total of 83 patients who consented to participate in the study were included from June to August, 2015. The case definitions and diagnostic criteria of surgical site infections were according to the guidelines on prevention of SSI by center for disease control and prevention and protocol on surveillance of SSI by European center for disease control and prevention^1,28^. Sampling was done by convenient sampling. Two wound swabs were collected aseptically from each patient using sterile cotton swabs by experienced laboratory personnel on the day of presenting with clinical SSI and before application of antiseptics. The swabs were immediately dipped into a sterile tube containing two - three drops of sterile normal saline as described by Mulu *et al*., 2012, and delivered to bacteriology laboratory at Mbarara University of science and technology (MUST) within five minutes of collection. Socio-demographic and clinical data was obtained from the patients’ files and by physical examination using structured and pretested questionnaire.

### Laboratory procedures

One of the swabs was immediately inoculated on to Blood agar, Chocolate agar and MacConkey agar (All Oxoid Ltd England). With exception of Chocolate agar that was incubated in increased carbon dioxide, all other inoculated agar plates were incubated aerobically at 35–37 °C for 24 hr. The plates were further re-incubated for up to 48 hours in case of no growth after 24 hours. The second swab was used for direct Gram staining to make a presumptive diagnosis. Identification of isolates was done using combination of colonial characteristics, Gram staining characteristics and conventional standard biochemical tests. Analytical profile index, API20E (BioMérieux) was used to retest bacterial isolates in cases where conventional identification methods could not identify the isolates.

Antimicrobial susceptibility testing for the isolated pathogen was performed using Kirby-Bauer disc diffusion method according to Clinical and Laboratory Standards Institute (CLSI)^29^. About 2–3 isolated colonies selected from a pure culture of blood agar plate were mixed in a tube containing 5 ml sterile saline to form a homogenous suspension. The suspension was adjusted to achieve a turbidity equivalent to 0.5 McFarland standards using a photometric device (Densimat, BioMérieux). Within about 15 minutes, a sterile cotton swab was dipped into the adjusted suspension and the excess fluid removed. The dipped swab was evenly inoculated on the surface of Müller-Hinton agar (Oxoid, Ltd, England) by streaking in three different planes rotating the plate approximately 60° each time and then the rim of the plate swabbed once. Selected antibiotic discs were place aseptically on the surface of the inoculated media after 5 minutes using sterile pair of forceps. The under listed BD BBL Sensi-Discs were used: Ceftriaxone (30 µg), Ciprofloxacin (5 µg), Gentamycin (10 µg or 30 µg), Sulfamethoxazole/Trimethoprim (25 µg), and Ampicillin (10 µg) for both Gram positive and negative bacteria, and Erythromycin (15 µg) and Oxacillin (1 µg) for Gram positive bacteria. Gentamycin (30 µg) was used for *Enterococcus species* to detect aminoglycoside resistance. The antibiotic discs were selected based on the availability and prescription frequency at the study site and CLSI guidelines (CLSI, 2012). The plates were inverted and incubated aerobically at 35–37 °C for 18–24 hours, after which diameter of zone of inhibition measured in millimetre and interpreted according to CLSI guidelines. Standard Control strains of *Escherichia coli* ATCC 25922 and *Staphylococcus aureus* ATCC 25923 as per CLSI guidelines were used for Gram negative and Gram positive organisms respectively to assure precision and accuracy of the test procedure and performance of the test materials. Multidrug resistance (MDR) was defined as non-susceptibility to at least one agent in three or more antimicrobial categories^30^. Because antibiotics used were of different categories, MDR meant resistance to three or more antibiotics tested.

### Data analysis

Data was entered into excel and then exported to be analyzed in SPSS version 16 software. Data was described as mean (±standard deviation) for age and as proportion for all categorical variables. Significance of relationship between dependent and independent variables was analysed using Chi-square test. A p-value of <0.05 was considered as statistically significant.

### Ethics approval and consent to participate

The study was approved by Mbarara University of Science and Technology research ethics committee. The research was performed in accordance with the ethical guidelines and regulations of the declaration of Helsinki. Informed consent was obtained from all participants and/or their parent/legal guardian.

## Data Availability

The datasets generated during and/or analysed during the current study are available from the corresponding author on reasonable request.

## References

[1] Mangram AJ (1999). Guideline for prevention of surgical site infection, 1999. Infection Control & Hospital Epidemiology.

[2] Dancer SJ, Stewart M, Coulombe C, Gregori A, Virdi M (2012). Surgical site infections linked to contaminated surgical instruments. Journal of Hospital Infection.

[3] Owens C, Stoessel K (2008). Surgical site infections: epidemiology, microbiology and prevention. Journal of Hospital Infection.

[4] Organization, W. H. *Global guidelines for the prevention of surgical site infection*. (World Health Organization, 2016). 27929621

[5] Anderson DJ, Sexton DJ, Kanafani ZA, Auten G, Kaye KS (2007). Severe surgical site infection in community hospitals: epidemiology, key procedures, and the changing prevalence of methicillin-resistant Staphylococcus aureus. Infection Control & Hospital Epidemiology.

[6] Seni J (2013). Antimicrobial resistance in hospitalized surgical patients: a silently emerging public health concern in Uganda. BMC research notes.

[7] Organization, W. H. Report on the burden of endemic health care-associated infection worldwide (2011).

[8] Allegranzi B (2011). Burden of endemic health-care-associated infection in developing countries: systematic review and meta-analysis. The Lancet.

[9] Nejad SB, Allegranzi B, Syed SB, Ellis B, Pittet D (2011). Health-care-associated infection in Africa: a systematic review. Bulletin of the World Health Organization.

[10] Weigelt JA (2010). Surgical site infections: causative pathogens and associated outcomes. American journal of infection control.

[11] Kitara D, Kakande I, Mugisa B, Obol J (2010). The postoperative complications prediction in Mulago Hospital using POSSUM scoring system. East and Central African Journal of Surgery.

[12] Lubega, A., Joel, B. & Justina Lucy, N. Incidence and etiology of surgical site infections among emergency postoperative patients in mbarara regional referral hospital, South Western Uganda. *Surgery research and practice* **2017** (2017). 10.1155/2017/6365172 PMC5266862 28168215

[13] Anguzu, J. & Olila, D. Drug sensitivity patterns of bacterial isolates from septic post-operative wounds in a regional referral hospital in Uganda. *African health sciences* **7** (2007). 10.5555/afhs.2007.7.3.148 PMC2269712 18052868

[14] Manyahi J (2014). Predominance of multi-drug resistant bacterial pathogens causing surgical site infections in Muhimbili National Hospital, Tanzania. BMC research notes.

[15] Dessalegn L, Shimelis T, Tadesse E, Gebre-selassie S (2014). Aerobic bacterial isolates from post-surgical wound and their antimicrobial susceptibility pattern: a hospital based cross-sectional study. E3 journal of medical research.

[16] Amatya J, Rijal M, Baidya R (2015). Bacteriological study of the postoperative wound samples and antibiotic susceptibility pattern of the isolates in BB hospital. *JSM*. Microbiology.

[17] Rao R, Sumathi S, Anuradha K, Venkatesh D, Krishna S (2013). Bacteriology of postoperative wound infections. Int J Pharm Biomed Res.

[18] Mawalla B, Mshana SE, Chalya PL, Imirzalioglu C, Mahalu W (2011). Predictors of surgical site infections among patients undergoing major surgery at Bugando Medical Centre in Northwestern Tanzania. BMC surgery.

[19] Mengesha RE, Kasa BG-S, Saravanan M, Berhe DF, Wasihun AG (2014). Aerobic bacteria in post surgical wound infections and pattern of their antimicrobial susceptibility in Ayder Teaching and Referral Hospital, Mekelle, Ethiopia. BMC research notes.

[20] Ameh EA (2009). Surgical site infection in children: prospective analysis of the burden and risk factors in a sub-Saharan African setting. Surgical infections.

[21] Giacometti A (2000). Epidemiology and microbiology of surgical wound infections. Journal of clinical microbiology.

[22] Amare B (2011). Postoperative surgical site bacterial infections and drug susceptibility patterns at Gondar University Teaching Hospital, Northwest Ethiopia. J Bacteriol Parasitol.

[23] Anthony A, Anthony I, Steve J (2010). Studies on multiple antibiotic resistant bacterial isolated from surgical site infection. Scientific Research and Essays.

[24] Gelaw A, Gebre-Selassie S, Tiruneh M, Mathios E, Yifru S (2014). Isolation of bacterial pathogens from patients with postoperative surgical site infections and possible sources of infections at the University of Gondar Hospital, Northwest Ethiopia. J Environ Occup Sci.

[25] Mulu W, Kibru G, Beyene G, Damtie M (2012). Postoperative nosocomial infections and antimicrobial resistance pattern of bacteria isolates among patients admitted at Felege Hiwot Referral Hospital, Bahirdar, Ethiopia. Ethiopian journal of health sciences.

[26] Rothe C, Schlaich C, Thompson S (2013). Healthcare-associated infections in sub-Saharan Africa. Journal of Hospital Infection.

[27] Sievert DM (2013). Antimicrobial-resistant pathogens associated with healthcare- associated infections summary of data reported to the National Healthcare Safety Network at the Centers for Disease Control and Prevention, 2009–2010. Infection Control & Hospital Epidemiology.

[28] Prevention, E. C. f. D. & Control. (European Centre for Disease Prevention and Control 2012).

[29] Wayne, P. Clinical and laboratory standards institute. Performance standards for antimicrobial susceptibility testing. (2011). 31339681

[30] Magiorakos AP (2012). Multidrug‐resistant, extensively drug‐resistant and pandrug‐resistant bacteria: an international expert proposal for interim standard definitions for acquired resistance. Clinical microbiology and infection.

